# Supplementary figures and images for: Embodied working memory during ongoing input streams
Source: PLoS One. 2021 Jan 5;16(1):e0244822. doi: 10.1371/journal.pone.0244822 (PMC7785253; doi:10.1371/journal.pone.0244822)

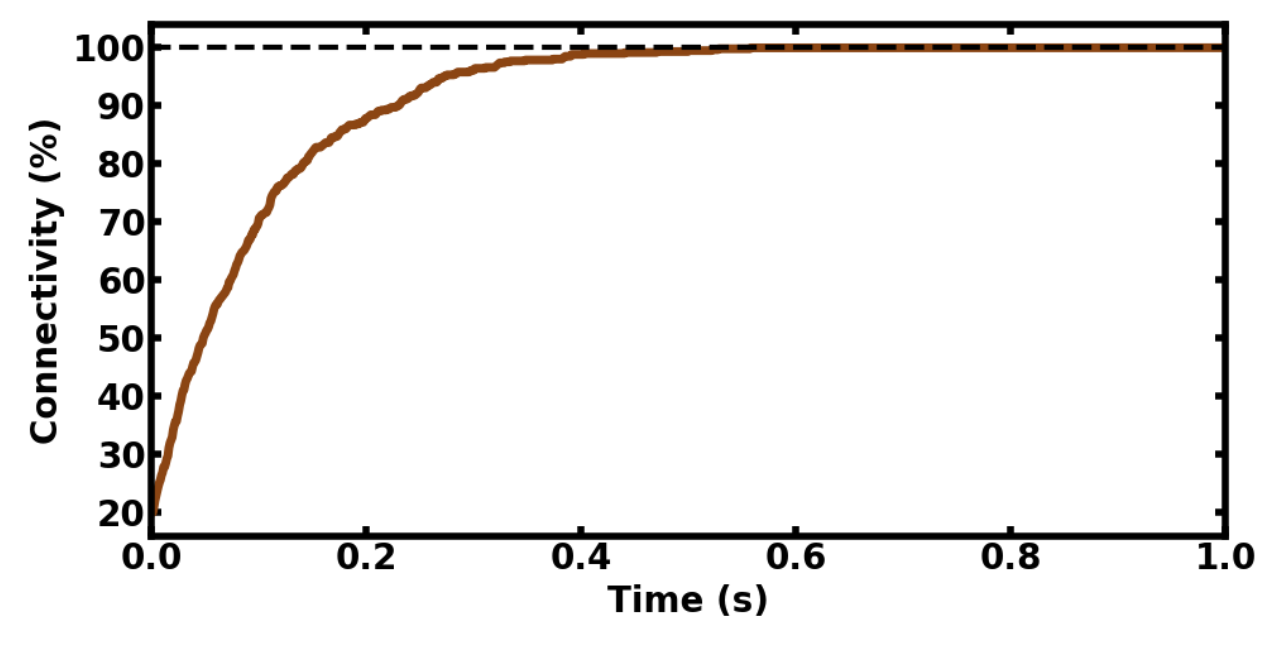

Supplement: S1 Fig — (TIF) [file pone.0244822.s001.tif]

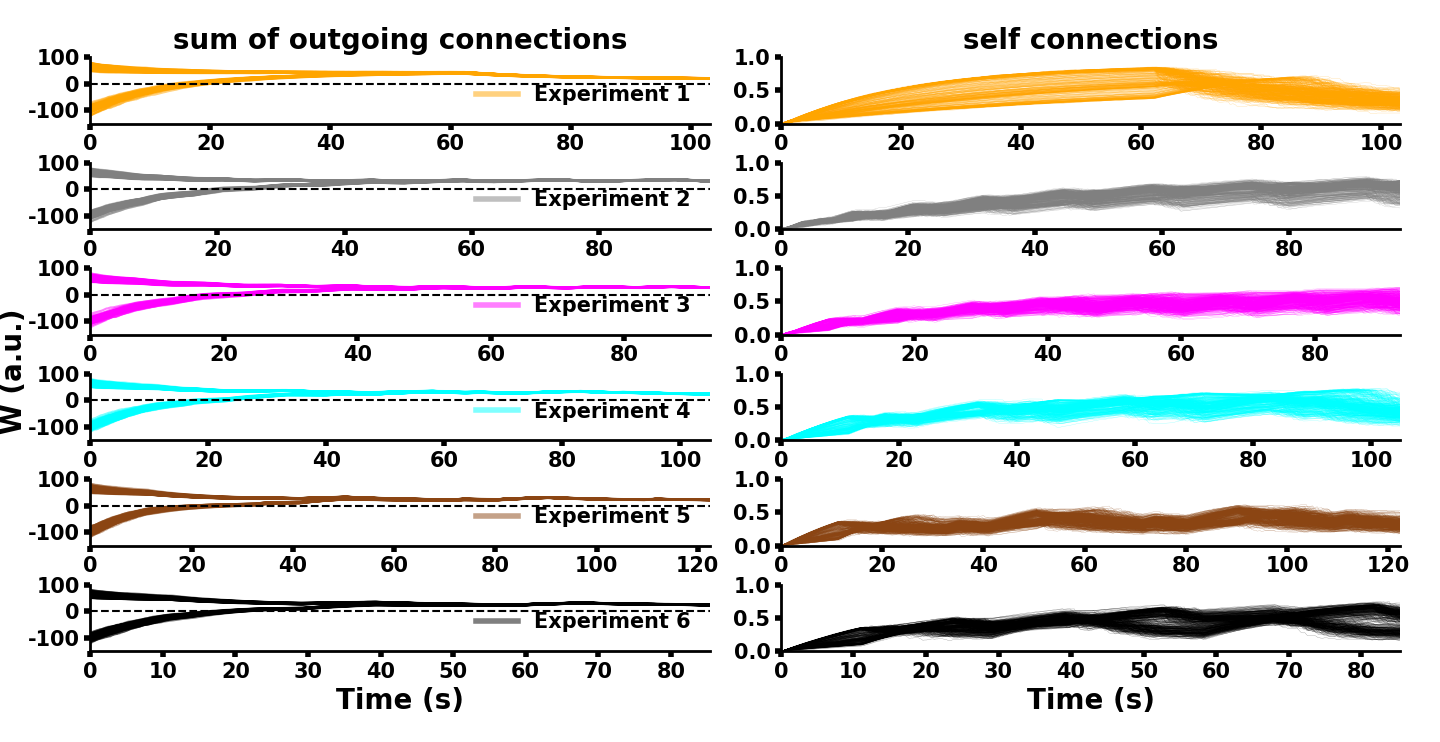

Supplement: S2 Fig — (TIF) [file pone.0244822.s002.tif]

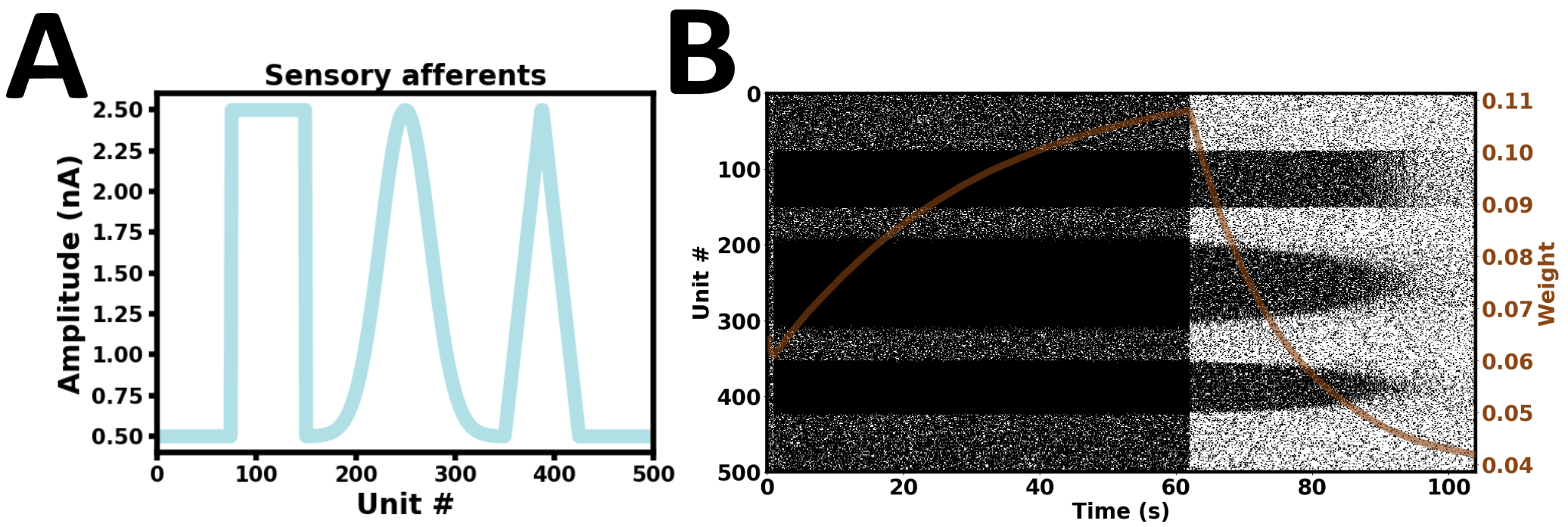

Supplement: S3 Fig — (TIF) [file pone.0244822.s003.tif]
